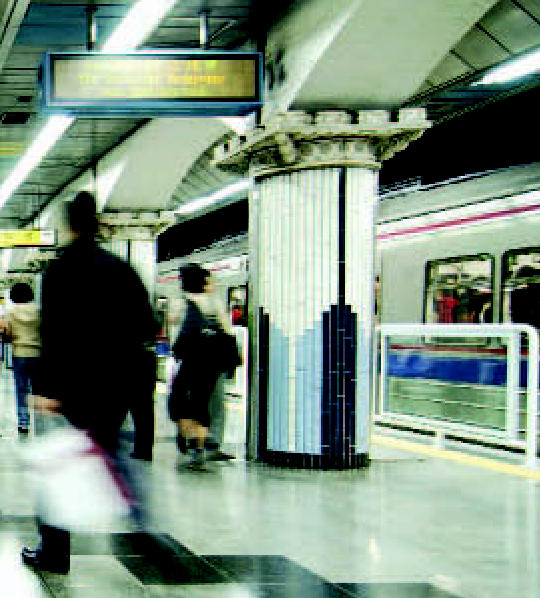# The Beat

**Published:** 2007-04

**Authors:** Erin E. Dooley

## Global Map of Malaria Risk

Scientists from the University of Oxford and the Kenya Medical Research Institute have just completed the first data-gathering stage of the Malaria Atlas Project, which will identify populations most at risk for malaria and predict the disease’s impact. Malaria data have been gathered from more than 3,000 communities in 79 countries. A final map will be generated using data from satellites, censuses, and other sources, with statistical methods filling in data gaps. Policy makers and funding agencies can utilize this information to better target resources. The open-access project is described in the December 2006 edition of *PLoS Medicine*.

**Figure:**
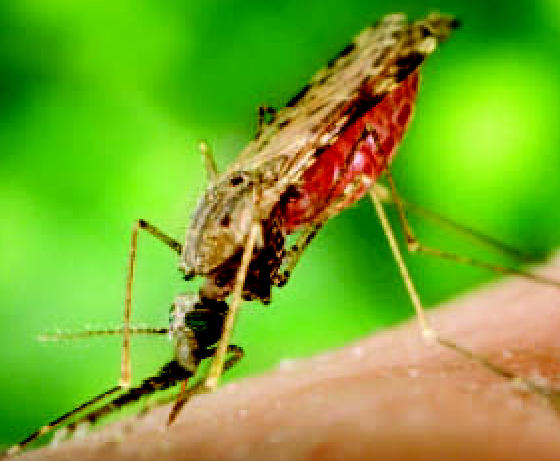


## China Top CO_2_ Producer by 2010

An International Energy Agency report issued in November 2006 estimates that China will overtake the United States as the largest producer of carbon dioxide by 2010, a decade sooner than earlier studies projected. A large percentage of the carbon dioxide emitted in the world comes from coal consumption. Seventy percent of China’s energy comes from coal, which is cheap and abundant in that country. China has stated it will try to limit coal production to 2.6 billion metric tons by 2010, but experts say this goal will probably not be met—the Chinese government is planning 500 new coal-fired power plants to meet the nation’s energy demands.

## Airline Rules Stir Up Controversy

In December 2006 the European Commission proposed new rules to regulate greenhouse gas emissions from the airline industry. Such emissions have increased 87% since 1990, and by 2020 are expected to more than double over present levels. The regulations place a cap on carbon dioxide emissions, with each airline receiving a set number of pollution allowances each year. Those using up their allowances must buy carbon credits from companies that still have them. As proposed, the rules will apply by 2012 to all flights to and from airports within the European Union. However, U.S. industry and government officials feel the rules violate international trade agreements and may fight the regulations if they are passed.

**Figure:**
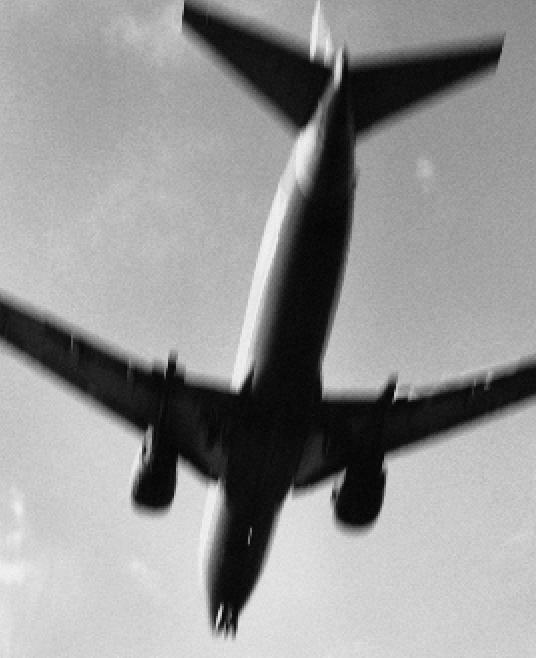


## The Sting of Climate Change

The number of people treated for jellyfish stings in Australia doubled to 26,000 between 2005 and 2006. An Australian jellyfish expert warns that the number of people stung each year could continue to rise as rising ocean temperatures may result in longer jellyfish seasons and larger jellyfish populations. Lisa-Ann Gershwin, an advisor to Surf Life Saving, also says that jellyfish, an ancient species, are “very good at taking advantage of changing conditions.” The nonstinging *Mnemiopsis leidyi*, for instance, thought to have been introduced to the Black and Caspian Seas via ballast water, has flourished to the point of decimating local ecosystems.

**Figure:**
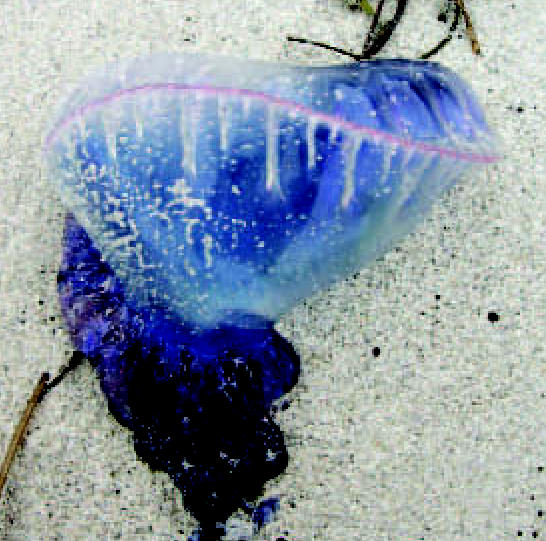


## Antimicrobial Nanoparticles to Be Regulated

The U.S. EPA announced in November 2006 that it would begin regulating consumer items made with nanoparticles of silver, the first time the agency has regulated any nanomaterial. Among the products made with these bactericidal nanoparticles are food-storage containers, air fresheners, shoe liners, and washing machines. Environmentalists worry that the growth of this technology may contribute to the killing off of beneficial bacteria and aquatic organisms, with adverse ramifications for human health. Only products that claim to kill germs will be affected by the rules.

## Asbestos Found in Korean Subways

Inspectors with the Seoul Metro subway in South Korea found asbestos in passenger-accessible sections of 17 of 30 subway stations they inspected in the winter of 2006. This follows a January 2004 *Environment International* study that found that Korean repair workers were being exposed to chrysotile and tremolite fibers in the subway. Asbestos was used in the construction of the stations to absorb noise and vibration from trains and is mainly present in ceiling tiles and insulation. Seoul Metro issued a statement on 22 January 2007 that it will remove the asbestos, though experts in Korea say the lack of trained asbestos removal specialists in the country will make it difficult to complete the job safely and effectively.

**Figure:**